# Body of evidence: integrating Eduard Pernkopf’s *Atlas* into a librarian-led medical humanities seminar

**DOI:** 10.5195/jmla.2017.223

**Published:** 2017-04

**Authors:** Keith C. Mages, Linda A. Lohr

## Abstract

**Background:**

Anatomical subjects depicted in Eduard Pernkopf’s richly illustrated *Topographische Anatomie des Menschen* may be victims of the Nazi regime. Special collections librarians in the history of medicine can use this primary resource to initiate dialogs about ethics with medical students.

**Case Presentation:**

Reported here is the authors’ use of Pernkopf’s *Atlas* in an interactive medical humanities seminar designed for third-year medical students. Topical articles, illustrations, and interviews introduced students to Pernkopf, his *Atlas*, and the surrounding controversies. We aimed to illustrate how this controversial historical publication can successfully foster student discussion and ethical reflection.

**Conclusions:**

Pernkopf’s *Atlas* and our mix of contextual resources facilitated thoughtful discussions about history and ethics amongst the group. Anonymous course evaluations showed student interest in the subject matter, relevance to their studies, and appreciation of our special collection’s space and contents.

## BACKGROUND

As repositories of historic primary source materials, medical library special collections are well suited to facilitate ethical discussions among medical students. One particular work with deep ethical implications is Eduard Pernkopf’s (1888–1955) anatomical atlas, *Topographische Anatomie des Menschen (Atlas),* which is a series of richly illustrated anatomical works that are infamous for the inclusion of Nazi holocaust victims depicted among its anatomical subjects [1, 2].

In 1997, libraries with copies of the *Atlas* received a letter from the University of Vienna, “Information for the Users of the Pernkopf-Atlas,” a one-page sheet that briefly describes Pernkopf’s connections to the Nazi regime. This letter voiced the possibility that victims of this regime may have been included among the *Atlas*’s illustrations [3]. It was left to the discretion of the library as to “whether and in what way” the work should be used. Our library withdrew its copies from the circulating collection and brought them to the History of Medicine Collection stacks, where they have been since. Having recently come across the Pernkopf volumes and the accompanying letter from Vienna, the authors were struck by the potential teaching opportunities about history and ethics that these resources provided.

Doctors, professors, and librarians have all weighed in on the Pernkopf debate, with individuals in each group espousing a variety of opinions and take-away messages. In their galvanizing letter to the editors of *JAMA* in 1996, Israel and Seidelman implored Pernkopf’s home institution, the University of Vienna’s Institute of Anatomy, to open a formal investigation to identify any “victims of the Nazi terror whose cadavers were…exploited by Pernkopf” [4]. Panush responded to Israel and Seidelman’s *JAMA* letter with one of his own, citing that such remnants of “Nazi medical legacy should be expunged from our legitimate professional heritage and literature” [5]. Williams, a professor of medical art and illustration, argued in an earlier piece for the *Atlas*’s value, stating that due to its connections to the Nazi regime, *Topographische Anatomie des Menschen* “will always be controversial…and never be acknowledged by some as the masterpiece it truly is” [6]. Meanwhile, anatomist and medical historian Hildebrant has advocated for the continued publishing of Pernkopf’s *Atlas,* calling for the addition of historical annotation throughout, thus teaching not only “anatomy, but also history and ethics” [7].

Professionals in the information sciences have also written on this topic. Librarian Michel C. Atlas authored a powerful article in the *Bulletin of the Medical Library Association* that provided an overview of the *Atlas,* its controversy, and the librarian’s role in the censorship of controversial materials [8]. Atlas strongly advocated for libraries to retain their copies of *Topographische Anatomie des Menschen,* suggesting that they add supplementary statements to catalog records and physical copies to alert readers to the historical and ethical issues at hand. Regardless of viewpoint, however, the Pernkopf *Atlas* clearly provides a powerful medium through which compelling discussions about history and ethics can be elicited.

## STUDY PURPOSE

Each year, the University of Buffalo’s Jacobs School of Medicine and Biomedical Sciences’ Center for Medical Humanities offers humanities coursework to third-year medical students. Students select from a variety of concurrent sessions, choosing the particular seminars that they wish to attend. Aware of these humanities offerings, the special collections librarians of the Robert L. Brown History of Medicine Collection approached the faculty administrator with the idea to lead a hands-on session on Pernkopf’s *Topographische Anatomie des Menschen.* Here, the authors describe our experience teaching an ethics-focused educational seminar to introduce medical students to this historical atlas and to facilitate the examination of relevant articles, photographs, book reviews, and interviews so that they might gain a deeper understanding of Pernkopf, his *Atlas,* and the controversy that surrounds both the man and his work. We aim to illustrate how this controversial historical publication can successfully foster student discussion and reflection on ethics.

## CASE PRESENTATION

In December 2015, the librarians of the Robert L. Brown History of Medicine Collection delivered a two-hour seminar session to a self-selected group of ten third-year medical students: “Ethics and an Anatomical Atlas: The Story of Eduard Pernkopf’s *Topographische Anatomie des Menschen.”* Prior to this session, the seminar’s contents and activities were submitted to and approved by the course’s supervising medical school faculty. The syllabus, course objectives, and required readings were made available via Blackboard, the university’s online course management system. Students were instructed to download the syllabus and come to the session having read two background articles, namely, Israel and Seidelman’s letter to the editors of *JAMA* and Hildebrandt’s “How the Pernkopf Controversy Facilitated a Historical and Ethical Analysis of the Anatomical Sciences in Austria and Germany,” an illuminating piece that provides a nuanced historical overview of Pernkopf’s life and *Atlas* as well as insight into subsequent investigations into his work at Vienna’s Institute of Anatomy (which were a direct result of Israel and Seidelman’s and others’ calls for action) [4, 7].

The seminar was held in the History of Medicine Collection reading room. Upon arrival, students were given a brief tour and overview of the collection. Students were next introduced to the *Topographische Anatomie des Menschen.* Toward this end, we made the first two English editions of the *Topographische Anatomie des Menschen* available [1, 2]. As students evaluated the physical copies of the books, they were oriented to particularly noteworthy illustrations as well as its publication history and contemporary critical reception. Discussions regarding the controversial nature of Pernkopf’s work were intentionally avoided at this point. We hoped the students might appreciate the *Atlases* as physical objects separated from any historical context, much as readers of the first English edition of the *Topographische Anatomie des Menschen* might have approached the work before the grim background of its anatomical subjects came to light.

After hands-on time with the books, students were advised to pay particular attention to the artists’ signatures found upon each illustration. While many of these signatures were quite mundane, others contain much more sinister details. Failing to discover the outliers through happenstance, the students were directed to turn to specific pages. On these panels, they encountered signatures featuring embedded swastikas and *Schutzstaffel (SS)* Bolts (for example, see Figure 265: Nerves and blood vessels in fossa carotica [9]). Following a period of group reflection, these visual remnants of Nazi ideology were used to connect the *Atlas* itself to the more contemporary debates regarding its place in academia.

Due to its powerful yet succinct format, we turned to analyze Israel and Seidelman’s letter to the editors of *JAMA.* At this point, we found that despite our original intention, more than half of the students had not accessed the readings before the seminar. As the piece was brief, a student was enlisted to read the letter aloud. Together, the group worked to identify the ethical issues presented in the letter. Issues of consent, autonomy, and cadaver rights were addressed.

Using photographs and text presented via PowerPoint, we next delivered an overview of Pernkopf’s life, his career at the University of Vienna’s Institute of Anatomy, and his evolving interest in and involvement with the Nazi regime. A detailed look at the procurement of cadavers after the *Anschluss* of 1938 (the annexation of Austria into the Third Reich) followed. For example, in February 1939, a decree from the minister of education of the German Reich proclaimed all bodies of executed prisoners were to be sent to the nearest department of anatomy. Estimates of the number and ethnicity of cadavers delivered to Pernkopf’s Institute of Anatomy were discussed (delivered cadavers of executed prisoners numbered in the thousands, although the number of Jewish descent and which of these bodies were featured in Pernkopf’s *Atlas* could not be readily ascertained) [7]. It is important to note that much of these data were only discovered fairly recently, again as a result of the investigation advocated by Israel and Seidelman. As much of this material was covered in the Hildebrandt text, which many of the students had not read, extra time was taken during this portion of the seminar to allow group reflection.

Students reunited after a short break to read Aharinejad and Carmichael’s “First Hand Accounts of Events in the Laboratory of Prof. Eduard Pernkopf” [10]. This article consisted of three interviews, each with a different professor who worked with Pernkopf at Vienna’s Institute of Anatomy. The questions that the interviewer asked are piercing, and some of the accounts are chilling. To maximize its affecting potential, students were not made aware of this article prior to the session. Again, student volunteers were procured to read the interviews. One student read aloud the interviewers’ questions, while another read the professors’ responses. After each of the three interviews, we facilitated group reflection and discussion. As each interviewed professor exhibited a different attitude toward Pernkopf and the Nazi-era Institute of Anatomy (ranging from dismissive, to saddened, to defensive), these readings were powerful catalysts for group discussion. Afterward, the group’s attention was guided back to the *Topographische Anatomie des Menschen,* a work clearly brilliant to the eye yet repulsive to the soul.

After viewing the *Atlas,* discovering its historical context, and reading firsthand accounts of Pernkopf, we asked the students what we should do with our copies of this resource. Before the students answered, we presented overviews of viewpoints from both sides of the debate: powerful quotes against its use were juxtaposed alongside those advocating for its preservation [11, 12]. A very short, anonymous survey with only one question was handed to each student: “What should we do with our copies of Pernkopf’s *Topographische Anatomie des Menschen?”* They were provided with six options to choose from:

- withdraw from holdings/destroy
- suppress usage; house in controlled stacks and allow usage only on a case-by-case basis
- undecided/neutral
- make available without caveat
- make available with appropriate historical caveat
- promote with appropriate historical caveat

The students filled these out quietly. Some took several seconds, carefully considering their responses. After we collected the responses, the group was asked if they wanted to hear the results aloud. All students answered affirmatively. Eight of the ten students believed the atlas should be made freely available at the library as long as historical contextual information was included for interested parties. Two students advocated for a higher level of promotion to actively tell the story of Pernkopf and his *Atlas.* None of the students considered withdrawing or suppressing the usage of Pernkopf’s work.

With this last activity completed, the seminar concluded. An anonymous online course evaluation, completed after the session, allowed student feedback. Students provided feedback on the overall strength of the session as well as specific constructive comments for session improvement. The comments that we received were largely positive (Table 1).

**Table 1:**
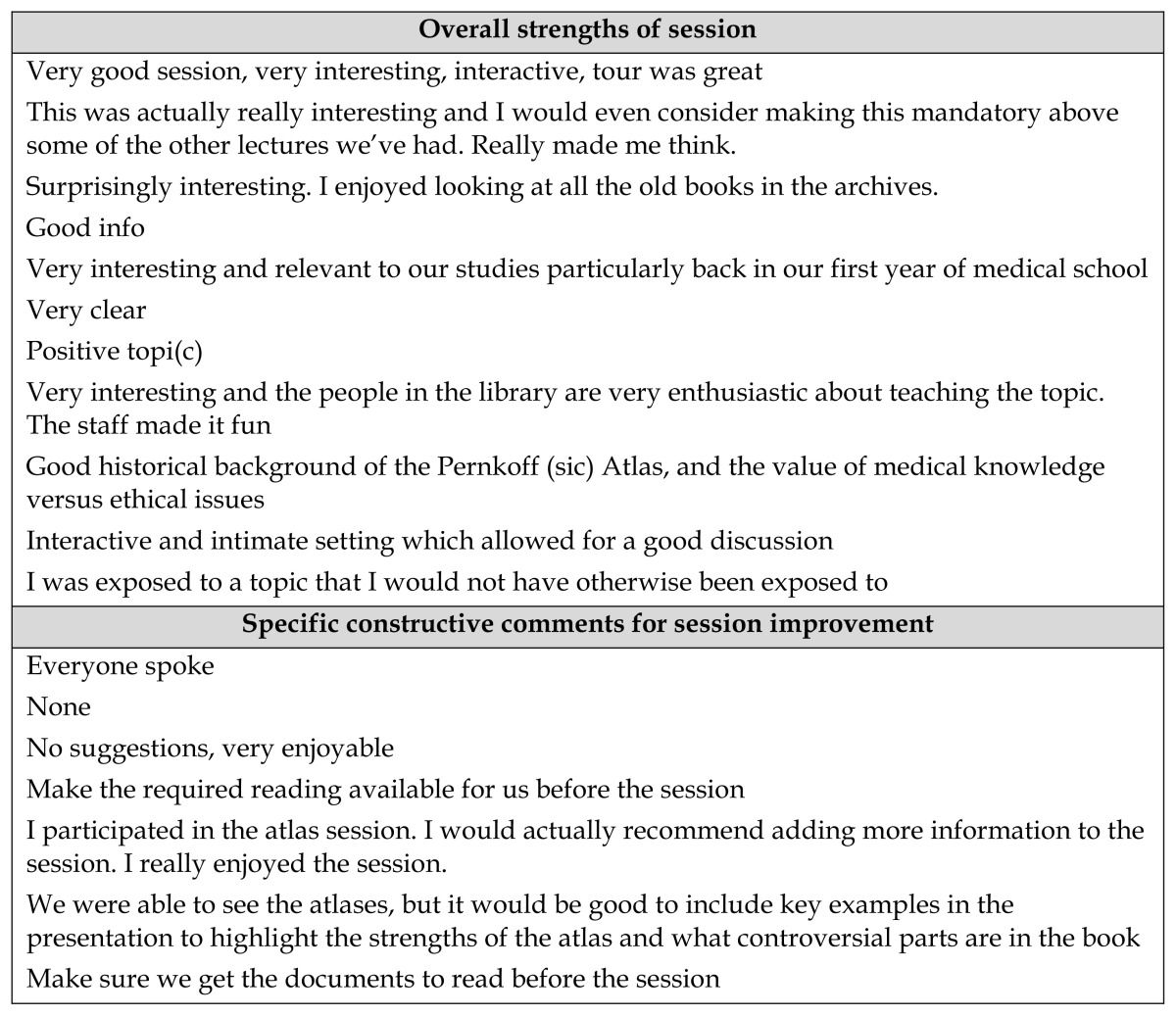
Student feedback

**Overall strengths of session**

Very good session, very interesting, interactive, tour was great
This was actually really interesting and I would even consider making this mandatory above some of the other lectures we’ve had. Really made me think.
Surprisingly interesting. I enjoyed looking at all the old books in the archives.
Good info
Very interesting and relevant to our studies particularly back in our first year of medical school
Very clear
Positive topi(c)
Very interesting and the people in the library are very enthusiastic about teaching the topic. The staff made it fun
Good historical background of the Pernkoff (sic) Atlas, and the value of medical knowledge versus ethical issues
Interactive and intimate setting which allowed for a good discussion
I was exposed to a topic that I would not have otherwise been exposed to

**Specific constructive comments for session improvement**

Everyone spoke
None
No suggestions, very enjoyable
Make the required reading available for us before the session
I participated in the atlas session. I would actually recommend adding more information to the session. I really enjoyed the session.
We were able to see the atlases, but it would be good to include key examples in the presentation to highlight the strengths of the atlas and what controversial parts are in the book
Make sure we get the documents to read before the session

## DISCUSSION

As instructors, we felt that this seminar was a successful undertaking. The physical copies of the *Atlas,* readings, photographs, and discussions provided a valuable mix of resources that evoked student interest and continuously facilitated discussion. Students articulated varying points of view and listened attentively to others’ comments. The signatures with embedded Nazi symbols, results of the inquiry into Pernkopf’s Institute of Anatomy, and interactive readings of the Aharinejad and Carmichael interviews were especially provocative and powerful components of the seminar. Each of these facets directly connected students to the extremism of Pernkopf’s era and readily promoted reflection and thoughtful discourse.

We were also encouraged by the results of our survey at the end of the course. All students appreciated the value of the Pernkopf *Atlas* and advocated for unfettered access as long as historical context was provided. At a minimum, we envision that all copies of *Topographische Anatomie des Menschen* should contain the University of Vienna’s “Information for the Users of the Pernkopf-Atlas,” the one-page sheet discussed earlier describing Pernkopf’s connections to the Nazi party [3].

The post-course online evaluations were also largely positive. The majority of students identified the seminar as “interesting.” From our perspective, interest arises from the presentation of novel knowledge. Students also referred to the experience as “interactive.” The seminar was specifically designed with this ideal in mind, as we believe it enhances learning and appreciation. Mention was also made of the History of Medicine Collection’s space, and this, too, was favorable. Participants described it as an intimate, enjoyable setting. Because we saw this seminar as an outreach opportunity meant to connect students with our collection, ourselves, and our resources, this finding was especially gratifying.

Although the seminar’s outcomes were largely satisfactory, some problems were encountered. Although the required readings had been available via the online course management system, many students did not access the required readings beforehand. We learned afterward that other concurrent humanities seminars also had this problem. Unfortunately, due to a miscommunication on the part of the administrative course faculty, not all students were made aware of the online content. We were assured that next year, students would be more explicitly directed to Blackboard to obtain course materials before the seminar dates. Student online evaluations noted that they indeed desired early access to seminar readings. We interpret such student evaluation comments as positive signs; if acquired beforehand, students felt our materials might have given them more perspective on Pernkopf and his *Atlas.* The online course evaluation also showed that students wanted more time to analyze the physical copies of the *Atlas.* During future seminars, the *Atlas* volumes will be left on the table so that students can peruse them throughout the session. We were indeed able to address these comments, as the History of Medicine Collection was invited to teach the Pernkopf seminar again during our medical school’s December 2016 medical humanities program.

Overall, this experience remains one of the most satisfying educational and outreach activities that the Robert L. Brown History of Medicine Collection has ever offered. We advise those who are interested in conducting similar seminars at their home institutions to make the time, make the connections, and take the risks. Pernkopf’s *Atlas* is just one of a number of historical texts that can contextualize ethics topics. Works such as Sir Francis Galton’s *Hereditary Genius: An Inquiry into Its Laws and Consequences* (1879), followed by his later more radical works on eugenics, can illuminate the slippery slope of scientific research. Also, archival images and textual documentation, available online from the National Archives, can recall the tragic realities faced by non-consenting African Americans during the Tuskegee Syphilis Study.

All medical practitioners will face ethical issues at some point in their careers. Exposure to complex ethical issues facilitates comprehension of patient vulnerability and physician fallibility, while also promoting awareness and understanding of both self and others. Experience discussing the nuanced realities present in ethical situations may help practitioners more objectively work through such circumstances when they do arise. Special collections librarians in the history of medicine are well positioned to facilitate dialogs about ethics, while these individuals are still enrolled in medical school, as their repositories house and promote the primary materials that enable such discussions. Historical books and artifacts foster frank, open group discussions, bridging the past to the present ethical issues at hand. The power of this connection was explicitly exhibited throughout our seminar session: Pernkopf’s *Topographische Anatomie des Menschen* provided students with perceptible insights into the past, a resonating ethics situation, and an appreciation for the place of history in contemporary medical education.
